# Improvement of Post-sympathectomy Raynaud’s Syndrome With Spinal Cord Stimulation

**DOI:** 10.7759/cureus.71340

**Published:** 2024-10-12

**Authors:** Kai Pan, Hongjie Jiang, Hemmings Wu, Junming Zhu, Jianmin Zhang

**Affiliations:** 1 Neurosurgery, The Second Affiliated Hospital of Zhejiang University, School of Medicine, Zhejiang Hangzhou, CHN; 2 Neurosurgery, Clinical Research Center of Neurological Disease of Zhejiang Province, Zhejiang Hangzhou, CHN; 3 Neurosurgery, The Second Affiliated Hospital of Zhejiang University, School of Medicine, Hangzhou Zhejiang, CHN; 4 Neurosurgery, Clinical Research Center of Neurological Disease of Zhejiang Province, Hangzhou Zhejiang, CHN

**Keywords:** finger necrosis, raynaud's phenomenon, spinal cord stimulation (scs), sympathectomy, vascular pain

## Abstract

Raynaud’s phenomenon is a vascular disorder, characterized by vasospasm-induced discoloration, numbness, and pain in the extremities. While pharmacological treatments and sympathectomy are commonly employed, many patients experience symptom recurrence, and effective therapies for refractory cases remain limited. This case study presents a 60-year-old male with severe Raynaud’s symptoms, including fingertip necrosis, unresponsive to pharmacotherapy and endoscopic thoracic sympathectomy. Despite initial symptom relief, the patient’s condition worsened, leading to finger necrosis. Spinal cord stimulation (SCS) was introduced as an alternative treatment, significantly reducing the patient's pain and improving blood flow to the affected areas. The mechanisms of SCS remain largely speculative, but it is believed to modulate the sympathetic nervous system, promoting vasodilation by releasing neuropeptides. This case highlights the potential of SCS as a therapeutic option for managing severe and recurrent Raynaud’s phenomenon, especially in patients unresponsive to conventional treatments.

## Introduction

Raynaud’s phenomenon is a vascular disorder that affects approximately 3%-5% of the population, manifesting as discoloration, numbness, and pain in the extremities, particularly the fingers and toes [1]. It is primarily caused by vasospasms, often triggered by cold exposure or stress, although its underlying pathophysiology remains inadequately understood [2]. While some young patients with Raynaud's phenomenon may experience spontaneous remission, severe cases present a 50% incidence of finger ulcers over a 10-year period, with up to 25% of patients requiring finger amputation. Approximately one-third of initially seropositive patients subsequently develop connective tissue disease [3]. The prognosis for these severe Raynaud's patients is determined by their underlying condition [4]. Generally, their quality of life is significantly impaired, and there have been documented cases of Raynaud's phenomenon associated with mixed connective tissue disease resulting in sepsis [5]. Current pharmacological management typically involves vasodilators and calcium channel blockers to improve circulation [6]. In more severe cases, sympathectomy is considered a primary intervention, while botulinum toxin injections have also shown promise in symptom management [7]. Some case reports suggest combining sympathectomy with botulinum toxin may be particularly effective in addressing complications such as digital necrosis associated with Raynaud’s phenomenon [8]. However, for patients with persistent or recurrent symptoms, especially those unresponsive to medical or surgical interventions, effective treatment options remain limited. In this case study, we describe a patient who experienced the recurrence of symptoms and progression to finger necrosis despite treatment with medication and sympathectomy. Following spinal cord stimulation (SCS), the patient showed marked symptomatic improvement, suggesting a potential new therapeutic avenue for refractory cases of Raynaud’s phenomenon.

## Case presentation

**Figure 1:**
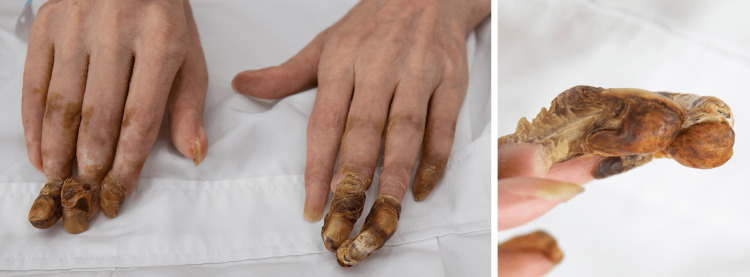
Signs of ischemia and necrosis of fingers at admission in 2020

**Figure 2:**
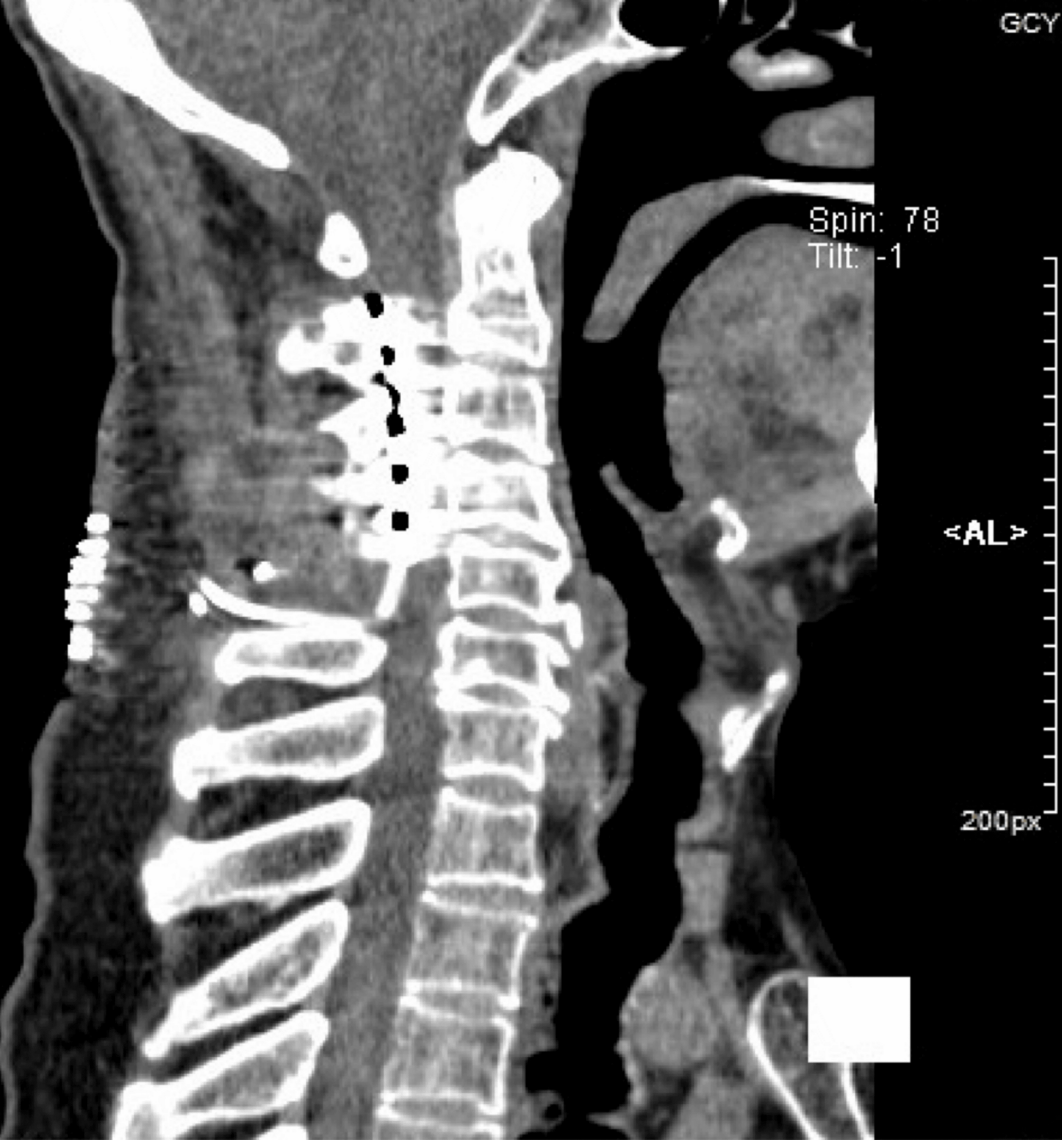
A postoperative review of the neck CT scan in the sagittal section reveals that a spinal cord stimulator was implanted at the C2 to C4 levels

**Figure 3:**
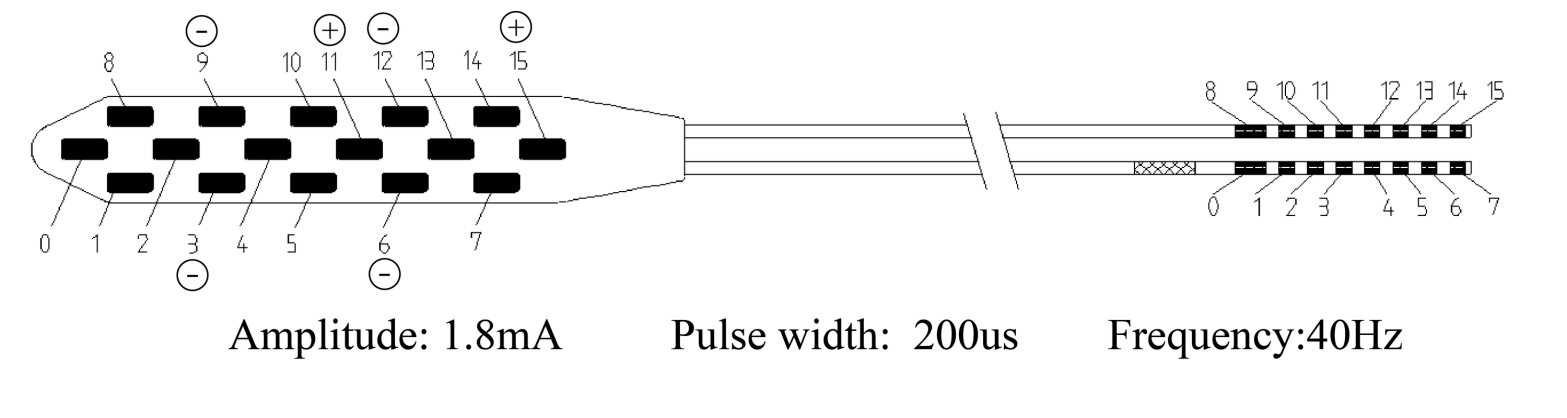
Stimulation parameters at implantation

A man in his 60s was referred to our center because of numbness and stinging pain in the fingertips of both hands for over five years. He has a history of diabetes but no family history of hereditary disorders. The first symptom onset was sudden and of no clear cause. Attacks of pain and numbness, with concomitant discoloration in the fingertips, were intermittent, and often triggered or exacerbated when exposed to cold. He had been given nifedipine and beraprost to promote circulation, and oxycodone for pain management, with limited clinical benefit. He received endoscopic thoracic sympathectomy at T2 to T4 level four years ago. Symptoms were relieved temporarily but returned within a couple of weeks. His condition progressed rapidly in the last 12 months, and necrosis developed in the distal phalanges of multiple fingers of both hands (Figure 1). Amputation was proposed but refused by the patient. On examination, the patient's Visual Analog Scale (VAS) score for pain was 10/10, the Patient Health Questionnaire-9 (PHQ-9) score was 27/27, and the Oswestry Disability Index (ODI) score was 49/50. Physical examination and laboratory results revealed no signs of carpal tunnel syndrome, rheumatic disease, or systemic sclerosis. The patient reported no history of smoking. Tinel’s Sign was negative. Antinuclear antibody level was normal. Rheumatoid factor and anticyclic citrullinated peptide were normal. Taken together, a diagnosis of Raynaud’s phenomenon was made. After careful evaluation, we proposed SCS to alleviate pain. A strip lead was placed epidurally covering C2 to C4 level under general anesthesia (Figure 2). Trial stimulation using an external stimulator reduced VAS score to 6, and the patient reported “a feeling of blood flow returning to fingertips.” A permanent stimulator was subsequently implanted (stimulation parameters are shown in Figure 3). After the surgery, the patient’s VAS score was 5, the PHQ-9 score was 13, and the ODI score was 42. The final follow-up occurred nine months post-discharge, at which time his VAS score was recorded as 4.

## Discussion

The central highlight of this case is the remarkable improvement in the patient's symptoms following SCS therapy, despite the recurrence of Raynaud’s symptoms after sympathectomy and the progression to fingertip necrosis. Sympathectomy typically aims to alleviate vasospasms by disrupting sympathetic nerve signals to the digital arteries, which theoretically reduces the overactivity of the sympathetic nervous system and promotes vasodilation [9]. However, sympathectomy's efficacy remains inconsistent, with a significant recurrence rate of Raynaud's symptoms post-surgery. This variability may stem from the lack of standardized surgical techniques and individualized patient responses, which has diminished its role as a preferred treatment option.

The mechanisms by which SCS exerts therapeutic effects in Raynaud’s phenomenon are not fully understood, though hypotheses focus on its potential modulation of the sympathetic nervous system. It is suggested that SCS may work by reducing sympathetic outflow and by antidromically activating sensory fibers, which, in turn, could release vasodilatory neuropeptides like calcitonin gene-related peptide (CGRP) [10]. The release of CGRP, known for its potent vasodilatory properties, may help counteract the vasospasm in affected areas, leading to improved circulation and symptom relief [11]. In this case, the improvement following SCS may indeed be linked to these neural and vasodilatory mechanisms, offering a therapeutic advantage that exceeds the outcomes of sympathectomy alone.

The clinical implications of SCS in treating Raynaud’s phenomenon are notable. While the procedure has demonstrated potential in alleviating symptoms, especially in refractory cases, it has yet to be incorporated into standard treatment guidelines. This may be due to the limited number of large-scale clinical trials and a lack of long-term outcome data. Nonetheless, several case reports and small studies have demonstrated that SCS can effectively manage Raynaud’s symptoms, particularly in severe cases resistant to conventional therapies [12-14]. Based on these reported cases, we recommend that, if feasible, future institutions conduct relevant clinical trials to further validate the therapeutic efficacy of SCS in treating Raynaud’s phenomenon and to assess its long-term prognosis. By sharing this case, we aim to raise awareness of SCS as a promising treatment for patients with limited options, encouraging its consideration in cases where traditional therapies fail.

## Conclusions

In light of the high recurrence rates associated with sympathectomy and the lack of consistently effective pharmacological treatments for refractory cases, SCS may represent a valuable therapeutic avenue. Further research, including controlled trials, is necessary to validate its efficacy and safety on a broader scale. This case contributes to the growing body of evidence supporting the potential role of SCS in managing severe and recurrent Raynaud’s phenomenon, highlighting the need for continued exploration into its mechanisms of action and clinical application.
